# Does Transcranial Direct Current Stimulation to Prefrontal Cortex Affect Mood and Emotional Memory Retrieval in Healthy Individuals?

**DOI:** 10.1371/journal.pone.0092162

**Published:** 2014-03-20

**Authors:** Helen M. Morgan, Nick J. Davis, R. Martyn Bracewell

**Affiliations:** 1 School of Natural Sciences & Psychology, Liverpool John Moores University, Liverpool, United Kingdom; 2 Department of Psychology, Swansea University, Swansea, United Kingdom; 3 School of Psychology and School of Medical Sciences, Bangor University, Bangor, United Kingdom; Cardiff University, United Kingdom

## Abstract

Studies using transcranial direct current stimulation (tDCS) of prefrontal cortex to improve symptoms of depression have had mixed results. We examined whether using tDCS to change the balance of activity between left and right dorsolateral prefrontal cortex (DLPFC) can alter mood and memory retrieval of emotional material in healthy volunteers. Participants memorised emotional images, then tDCS was applied bilaterally to DLPFC while they performed a stimulus-response compatibility task. Participants were then presented with a set of images for memory retrieval. Questionnaires to examine mood and motivational state were administered at the beginning and end of each session. Exploratory data analyses showed that the polarity of tDCS to DLPFC influenced performance on a stimulus-response compatibility task and this effect was dependent on participants’ prior motivational state. However, tDCS polarity had no effect on the speed or accuracy of memory retrieval of emotional images and did not influence positive or negative affect. These findings suggest that the balance of activity between left and right DLPFC does not play a critical role in the mood state of healthy individuals. We suggest that the efficacy of prefrontal tDCS depends on the initial activation state of neurons and future work should take this into account.

## Introduction

The prefrontal cortex appears to play an important role in affect and emotional processing [1]. Based on the idea that hemispheric asymmetries in prefrontal activity play a role in emotion regulation [2], [3], and findings of suppressed left prefrontal activity in depressed patients [4], [5], several attempts have been made to treat depression by altering the balance of activity between left and right prefrontal brain regions using transcranial direct current stimulation (tDCS) [6]. This technique uses low electrical current delivered to the scalp by two electrodes (the anode and cathode). Cortical excitability is increased under the anode and decreased under the cathode, and 10 minutes of stimulation can produce neural and behavioural effects lasting for up to 40 minutes [7]. Consecutive sessions of tDCS may produce effects on task performance lasting for several weeks; for example motor skill acquisition was enhanced following five sessions of anodal tDCS on consecutive days, and this improvement was still evident three months later [8].

However, controlled clinical studies of the efficacy of tDCS in the treatment of depression have shown mixed results, with some studies showing improvement of depression [9]–[12] and others showing no improvements [13]–[15] following consecutive days of anodal tDCS to left dorsolateral prefrontal cortex (DLPFC) compared to sham (“placebo”) tDCS. In addition, it is not clear whether any observed effects of tDCS on depression are specific to the direction of current flow. That is, studies involving depressed patients have only applied tDCS in the polarity thought to be therapeutic; while it is understandable that clinicians would wish to avoid using an electrode montage that may potentially worsen a person’s depression state, it remains unclear whether mood could be worsened as well as improved by tDCS.

Research using healthy individuals is important because it allows the study of prefrontal tDCS effects on mood in a more homogenous sample, and allows the possibility to manipulate mood in both directions. As well as using subjective self-report measures of emotional state (e.g. questionnaires), mood can be measured by examining performance on cognitive tasks that require processing of emotional information [16]. For example, memory retrieval of emotional material is more successful in moods that are congruent with the emotional content of the memories [17]. However, there has been very little work examining the effects of prefrontal tDCS on mood and processing of emotional information in healthy individuals, and the results of this work are inconsistent. Several studies have found no effects of prefrontal tDCS on mood, measured by self-report [18]–[24], and there have been mixed results from studies which have used cognitive tasks to measure mood indirectly [21]–[23], [25], [26]. There is also some evidence to suggest that the effects of prefrontal tDCS on cognitive tasks may depend on participants’ motivational bias in relation to the task [27], [28].

Previous studies into the effects of prefrontal tDCS on emotional state have used large tDCS electrodes (25–35 cm^2^) with a relatively low current density (0.029–0.057 mA/cm^2^) [20]–[26]. Models of current flow in tDCS suggest that the use of smaller electrodes results in increased focality and current density for a given current [29]. In support of these models, increasing current density and focality by reducing electrode size while holding current strength constant has been shown to increase the effects of tDCS on the excitability of motor cortex [30]. Therefore, it is possible that some of the negative results observed in previous work are due to insufficient focality and/or current density over DLPFC.

The present study aimed to determine whether the polarity of tDCS over left and right prefrontal cortex influences mood, as measured by memory retrieval of emotional material, in a relatively homogenous sample of healthy individuals, using a higher current density and smaller electrodes than those used in previous work.

## Materials and Methods

### Participants

18 right handed students of Bangor University (9 male), aged 19 to 30 (mean age 23.2) participated in return for money. None of the participants had a history of neurological or psychiatric disorders and they were not taking any psychoactive medication. The study was conducted in accordance with the British Psychological Society’s Code of Ethics and Conduct [31], and participants were free to withdraw from the study at any time without financial penalty. Participants provided written informed consent to the study by signing a printed consent form. The study and consent procedures received ethical approval from the Bangor University School of Psychology Ethics Committee.

### Stimuli and Procedure

The Positive Affect Negative Affect Schedule (PANAS) is a questionnaire consisting of 20 items designed to separately assess positive and negative mood [32]. Each item is rated on a five-point scale, with a rating of 1 indicating a low level (“very slightly or not at all”) and a rating of 5 indicating a high level (“extremely”) of each mood state. The sum of ratings for the 10 items representing a given mood is taken as a measure of the participant’s level of that mood state. The questionnaire of Motivational States [33] consists of 15 questions, each rated on a 5 point scale, designed to measure an individual’s motivational state in relation to the task. Both questionnaires have been shown to have high internal reliability (Cronbach’s α>0.8) and high construct validity with respect to other measures [32]–[34].

For each participant, the stimuli for the memory task were randomly selected from 462 images (154 neutral, 154 positive, and 154 negative) from the International Affective Picture System (IAPS) [35]. The valence of these images, rated on a scale from 1 (low, or negative, valence) to 9 (high, or positive, valence), ranged from 1.4 to 3.6 (mean = 2.8) for the negative images, 4.1 to 6.1 (mean = 5.0) for the neutral images, and 6.7 to 8.7 (mean = 7.6) for the positive images (35). The positive and negative image sets contained equal numbers of images depicting animals (34), objects (28), landscapes (21), and people (71), whereas the neutral images contained fewer animals (15), more objects (49), and similar numbers of landscapes (22) and people (76).

Participants were seated with their eyes 60 cm from a 19 inch TFT monitor, and the stimuli were sized to fill the screen. During the memory encoding phase, 50 positive, 50 negative, and 50 neutral images were presented in a random order. Each image was presented for 3 seconds and there was a 2 second interval between images. Participants were instructed to memorise each image. The memory retrieval phase contained 25 “old” images that had been presented during the encoding phase and 25 “new” images that were not included in the encoding phase for each level of valence (positive, negative, and neutral). Participants were instructed to respond “new” or “old” to each image using the index and middle fingers of their right hand on keys “M” and “N” of the computer keyboard. After the response the image was followed by a screen which asked participants to rate their confidence in the accuracy of their response on a scale from 1 (very unconfident) to 5 (very confident) using the number keys on the computer keyboard. The memory retrieval task lasted for approximately 10 minutes.

During the application of tDCS, participants performed a stimulus-response compatibility “filler task” in which blue or red circles were presented on the left and right of fixation, and participants had to respond to the blue circle with the left hand (key “A”) and to the red circle with the right hand (key “L”). There were 80 trials; on half the trials the location of the circle was incompatible with the responding hand and on the other half of trials it was compatible. The purpose of the filler task was to keep participants in a stable mood during tDCS by preventing them from having thoughts or conversations with the experimenter that may have altered their mood state. In addition, any effects of tDCS on this task would suggest that tDCS did indeed modulate prefrontal excitability.

Participants first completed the motivational state questionnaire, followed by the PANAS questionnaire. They then completed the encoding phase of the memory task, after which tDCS was applied for 12 minutes. During the tDCS participants first completed the filler task, then they received instructions for the retrieval phase of the memory task. Participants then completed the retrieval phase of the memory task, followed by the PANAS questionnaire. Each participant completed two experimental sessions, separated by at least a week. Half of the participants received right anodal/left cathodal (RA/LC) stimulation in the first session and left anodal/right cathodal (LA/RC) stimulation in the second session. The other half of participants received the sessions in the opposite order. A sham tDCS session was not included, as we were primarily interested in comparing opposite polarities of stimulation. Furthermore, sham tDCS causes significantly less skin sensation than active tDCS at current densities above 0.04 mA/cm^2^ [36], [37] and participants can reliably differentiate sham from active tDCS at a current density of 0.057 mA/cm^2^ [38]. For each participant, the memory task contained different images for each session to avoid carry-over effects between sessions.

### tDCS

Transcranial direct current was delivered using a Magstim DC+ (Whitland, Dyfed, Wales) constant current stimulator and rubber electrodes placed in sponges saturated with saline. The electrodes were circular with an area of 9 cm^2^ and were placed bilaterally over electrode positions F3 and F4 of the 10–10 system [39], corresponding to left and right DLPFC [40]. tDCS was applied at an intensity of 1 mA for 12 minutes (current density 0.11 mA/cm^2^). To minimise skin sensation, the current was ramped up and down for an additional 15 seconds at the beginning and end of stimulation. Participants reported non-painful skin sensations throughout the duration of tDCS.

### Outcome Parameters

The main outcome parameter was emotional memory retrieval, measured by performance on the memory task. The other outcome parameters were self-reported mood, measured by the PANAS, and performance on the filler task. We also performed exploratory analyses of the effects of motivational state and gender on these outcome parameters.

### Data Analysis

For the main outcome parameter, emotional memory retrieval, mean response times (RTs) and confidence ratings for correct-response trials were submitted to 2 (tDCS polarity)×3 (valence)×2 (new/old) repeated-measures ANOVAs. We calculated accuracy at discriminating between old and new pictures using A′ as a measure of signal detection sensitivity [41]. A′ was calculated using the following formula: A′ = 0.5+[(H–FA)×(1+H–FA)]/[4×H×(1–FA)] where H is hit rate (proportion of “old” images correctly identified as old) and FA is false alarm rate (proportion of “new” images incorrectly identified as old). If FA>H the following formula is used: A′ = 0.5−[(FA−H)×(1+FA−H)]/[4×FA×(1−H)]. A′ of 0.5 indicates chance performance and A′ of 1 indicates perfect performance. A′ was used because it is more robust than d′ against violations of the assumption that the hypothetical noise and signal plus noise distributions have equal variances [41], [42]. A′ scores for the memory retrieval task were submitted to a 2 (tDCS polarity)×3 (emotion) repeated-measures ANOVA. Our sample size of 18 participants was based on calculations showing that this sample would be sufficient to detect a medium effect size (Cohen’s d = 0.5) for the interaction between tDCS polarity and valence with a power level of 0.8 [43].

To examine effects of tDCS on self-reported mood, scores for positive and negative PANAS items were submitted to 2×2 ANOVAs with the factors tDCS polarity and time (pre-tDCS vs post-tDCS). For the filler task, mean RTs for correct-response trials and accuracy (percentage correct) were submitted to 2×2×2 ANOVAs with the factors tDCS polarity, stimulus location (left or right), and response location (left or right).

We also performed additional exploratory analyses by separately adding the factors motivational state and gender to the analyses described previously. The effect of motivational state was examined by performing a median split of participants for each of the tDCS sessions. Participants’ motivational states were consistently high or low for both of the sessions, with the exception of two participants, who were excluded from this analysis because they were assigned to the high motivation group in one session and the low motivation group for the other session.

Finally, we have included Bayesian analyses as a complement to the conventional null-hypothesis statistical tests which force a binary choice between the null and the alternative hypotheses. Bayesian methods are more sensitive to the evidence for the different positions; the relative evidence for a given hypothesis is represented by the Bayes factor [44]. Note that whereas conventional tests such as ANOVA examine the significance of individual effects, Bayesian analysis involves building and comparing different models and does not consider effects separately. For example, to examine an interaction the main effects must also be included in the model. The best fitting model is the one with the highest Bayes Factor. A Bayes Factor greater than 3 or less than 0.33 is assumed to represent substantial evidence for the null or the alternative hypothesis, whereas a Bayes Factor between 0.33 and 3 is considered weak evidence [45]. Bayesian analysis takes into account the prior probability of each possible outcome; in our analyses we used non-informative (objective) priors. We used the BayesFactor package for R (http://www.R-project.org), which implements the methodology outlined in Rouder et al. [46].

## Results

### Memory Retrieval

Results for the memory retrieval task are shown in Table 1. The RT analysis found a significant main effect of valence, F(2, 34) = 12.3, p<.001, η_p_^2^ = 0.42; post-hoc tests (Bonferroni-corrected) revealed that RTs were slower for negative images compared to neutral images (p = .005) and positive images (p<.001). RTs were significantly faster to old images compared to new images, F(1, 17) = 29.8, p<.001, η_p_^2^ = 0.64. There was also an interaction between the valence of an image and whether the image was new or old, F (2, 34) = 4.5, p = .02, η_p_^2^ = 0.21. Follow-up ANOVAs revealed that the effect of valence on RTs was only significant for new images, F(2, 34) = 14.8, p<.001, η_p_^2^ = 0.47, not for old images, F(2, 34) = 1.9, ns, η_p_^2^ = 0.10. There was no main effect of tDCS polarity, F(1, 17) = 0.8, ns, η_p_^2^ = 0.05, and no other interactions were significant (all Fs <1.5, all p’s>.2, all η_p_^2^<0.08). Analysis of confidence ratings found that participants were more confident for “old” responses compared to “new” responses, F (1, 17) = 22.6, p<.001, η_p_^2^ = 0.57. There were no main effects of tDCS polarity, F (1, 17) = 1.2, ns, η_p_^2^ = 0.07, or valence, F (2, 34) = 0.3, ns, η_p_^2^ = 0.02, and there were no significant interactions (all Fs <2.0, all p’s>.1, all η_p_^2^<0.11). This analysis found no significant effects of tDCS polarity, F(1, 17) = 0.05, ns, η_p_^2^ = 0.003, or valence, F(2, 34) = 0.4, ns, η_p_^2^ = 0.02, and no interaction, F(2, 34) = 0.03, ns, η_p_^2^ = 0.002.

**Table 1:** Mean response time (RT), accuracy (A′), and confidence rating (CR) for positive, negative, and neutral images.

		LA/RC			RA/LC		
		Negative	Neutral	Positive	Negative	Neutral	Positive
RT	New	1380 (88)	1249 (70)	1298 (85)	1325 (64)	1232 (50)	1184 (51)
	Old	1183 (81)	1149 (75)	1135 (69)	1144 (41)	1115 (48)	1111 (59)
CR	New	4.22 (0.13)	4.22 (0.13)	4.19 (0.15)	4.33 (0.11)	4.34 (0.11)	4.37 (0.09)
	Old	4.73 (0.07)	4.72 (0.06)	4.72 (0.07)	4.76 (0.06)	4.75 (0.06)	4.71 (0.05)
A′		0.94 (0.01)	0.93 (0.01)	0.93 (0.01)	0.94 (0.01)	0.93 (0.01)	0.93 (0.01)

The Bayesian analysis of RTs showed that the best fitting model (i.e. the model with the highest Bayes Factor - BF) was the main effects model (BF>200). The model of the interaction between valence and tDCS polarity supported the null hypothesis (BF = 0.23). The best fitting model for confidence ratings was the model that only included the main effect of new/old (BF>200), whereas the model of the interaction between valence and tDCS polarity again supported the null hypothesis (BF <0.01). For accuracy (A′) data, the best fitting model was the main effect of polarity (BF = 0.21) and a model of the interaction also supported the null hypothesis (BF <0.01).

### PANAS

Results for the PANAS questionnaire are shown in Table 2. Analysis of positive mood found no main effect of tDCS polarity, F(1, 17) = 0.006, ns, η_p_^2^<0.001 or time, F(1, 17) = 0.08, ns, η_p_^2^ = 0.005, and no interaction, F(1, 17) = 1.5, ns, η_p_^2^ = 0.08. Analysis of negative mood revealed a significant effect of time, F(1, 17) = 5.4, p = .03, η_p_^2^ = 0.24, with a decreased level of negative affect post-tDCS compared to pre-tDCS. However, there was no main effect of tDCS polarity, F(1, 17) = 0.4, ns, η_p_^2^ = 0.02, and no interaction, F(1, 17) = 0.03, ns, η_p_^2^ = 0.86. Further analyses suggested that the decrease in negative affect after tDCS was due to a decrease in scores on the nervousness item, *t*(17) = 2.5, *p* = .02, *d* = 0.59, whereas the decreases on the other negative items did not reach significance.

**Table 2:** Mean scores before (pre) and after (post) tDCS for the PANAS. Standard errors are shown in parentheses.

	LA/RC		RA/LC	
	Pre tDCS	Post tDCS	Pre tDCS	Post tDCS
Positive affect	29.8 (1.4)	28.5 (1.7)	28.9 (2.0)	29.7 (2.1)
Negative affect	11.1 (0.6)	10.4 (0.2)	11.4 (0.5)	10.7 (0.3)

The corresponding Bayesian analysis showed that a model containing only the main effect of time was preferred for both positive mood (BF = 0.25) and negative mood (BF = 1.18). The models containing the interaction between time and tDCS polarity supported the null hypothesis for positive (BF = 0.03) and negative (BF = 0.12) mood, consistent with the classical ANOVA analysis which also showed no changes in mood after tDCS.

### Filler Task

Results for the filler task are shown in Table 3. The RT analysis found a significant interaction between stimulus location and response location, F (1, 17) = 24.3, p<.001, η_p_^2^ = 0.59, showing the typical stimulus-response compatibility effect [47]. That is, for left hand responses RTs were faster when the stimulus appeared on the left compared to the right, whereas for right hand responses RTs were faster when the stimulus appeared on the right compared to the left. No other main effects or interactions were significant (all Fs <1.9, all p’s>.1, all η_p_^2^<0.1). The accuracy analysis found significantly more correct responses when stimuli appeared on the right compared to the left, F (1, 17) = 5.5, p = .03, η_p_^2^ = 0.24. There were no main effects of tDCS polarity, F (1, 17) = 0.04, ns, η_p_^2^ = 0.002, or response location, F (1, 17) = 0.4, ns, η_p_^2^ = 0.02, and no interactions were significant (all Fs <0.5, all p’s>.4, all η_p_^2^<0.03).

**Table 3:** Mean response time (RT) in milliseconds and accuracy (percentage correct) for the filler task as a function of target location (left or right) and response location (left hand or right hand). Standard errors are shown in parentheses.

	Target location	Left		Right	
	Response location	Left	Right	Left	Right
LA-RC	RT	491 (20)	507 (23)	519 (24)	479 (20)
	% correct	95.3 (1.3)	94.4 (1.6)	95.6 (1.6)	96.7 (1.1)
RA-LC	RT	482 (20)	500 (23)	502 (21)	470 (21)
	% correct	95.6 (1.1)	93.9 (1.3)	96.1 (1.2)	95.8 (1.4)

For RTs the best fitting Bayesian model was the interaction between stimulus location and response location (BF = 41). By contrast, the model that included tDCS polarity in the interaction was not a good fit (BF = 0.01). In terms of accuracy, the best fitting Bayesian model was the main effect of stimulus location (BF = 0.57), and the model describing the three-way interaction was a poor fit (BF <0.01).

### Motivational State

Overall motivational state scores varied between 26 and 49 (mean = 37) in the LA-RC session and between 23 and 50 (mean = 36) in the RA-LC session. For the memory task, there were no main effects or interactions involving motivational state on RT, confidence ratings, or accuracy (all F’s <2.0, all p’s>.1, all η_p_^2^<0.12).

For the PANAS, there was a significant main effect of motivational state on positive affect, F (1, 14) = 7.9, p = .01, η_p_^2^ = 0.36, with higher scores in the high motivation group compared to the low motivation group. However, motivational state did not interact with any other variable (all Fs <3.0, all p’s>.1, all η_p_^2^<0.18), and there were no effects of motivational state on negative affect (all Fs <0.4, all p’s>.5, all η_p_^2^<0.03).

For the filler task, the RT analysis revealed an interaction between tDCS polarity, stimulus location, and motivational state, F (1, 14) = 5.8, p = .03, η_p_^2^ = 0.29, and an interaction between stimulus location, response location, and motivational state, F (1, 14) = 7.2, p = .02, η_p_^2^ = 0.34. Further analyses showed a significant interaction between tDCS polarity and stimulus location in the high motivation group, F (1, 7) = 9.6, p = .02, η_p_^2^ = 0.58, with faster RTs to targets on the right compared to the left during LA/RC tDCS, and faster RTs to targets on the left compared to the right during RA/LC tDCS, whereas the interaction between stimulus location and response location did not reach significance, F (1, 7) = 3.4, p = .1, η_p_^2^ = 0.33. By contrast the low motivation group showed a significant interaction between stimulus location and response location, F (1, 7) = 31.1, p = .001, η_p_^2^ = 0.82 (i.e. a significant stimulus-response compatibility effect), but no interaction between tDCS polarity and stimulus location, F (1, 7) = 1.5, ns, η_p_^2^ = 0.17. Bayesian analysis of RTs showed that the preferred model was the one that included both the interaction between stimulus location and response location and the interaction between motivational state and tDCS polarity (BF>200). The accuracy analysis found a marginally significant interaction between tDCS polarity and motivational state, F (1, 14) = 4.5, p = .05, η_p_^2^ = 0.24, with higher accuracy during LA/RC tDCS in the low motivation group and higher accuracy during RA/LC tDCS in the high motivation group. There were no other effects of motivational state in the accuracy analysis (all Fs <2.2, all p’s>.1, all η_p_^2^<0.14). The best fitting Bayesian model of the accuracy data included interactions between motivational state and response location, motivational state and stimulus location, tDCS polarity and response location, and tDCS polarity and stimulus location (BF = 9.5).

### Gender Effects

For the emotional memory task there was no main effect of gender on RTs and gender did not interact with any other variable (all Fs <1.3, all p’s>.3, all η_p_^2^<0.08). There were also no significant effects of gender on memory accuracy (all Fs <2.9, all p’s>.1, all η_p_^2^<0.16) or confidence ratings (all Fs <1.6, all p’s>.2, all η_p_^2^<0.09). Bayesian analysis showed that the preferred model for RTs was still the main effects model that did not include the effect of gender (BF>200). A model of the interaction between gender, valence, and tDCS polarity supported the null hypothesis (BF <0.01). The preferred model for confidence ratings was again the main effect of new/old (BF>200), and the model of the interaction between gender, valence, and tDCS polarity supported the null hypothesis (BF <0.01). For accuracy scores, the preferred model was the interaction between tDCS polarity and gender, although the evidence for this model was weak (BF = 1.1), and the model of the interaction between gender, valence, and tDCS polarity supported the null hypothesis (BF <0.01).

For the PANAS, there were no interactions containing gender or main effects of gender on positive affect (all Fs <0.7, all p’s>.4, all η_p_^2^<0.04). Females scored slightly higher than males on negative affect, F(1, 16) = 3.5, p = .08, η_p_^2^ = 0.18, and there was a marginally significant interaction between time and gender, F(1, 16) = 3.5, p = .08, η_p_^2^ = 0.18; post-hoc tests showed increased negative affect for females compared to males before tDCS (p = 0.1), but not after tDCS (p = 0.2), but these differences were not significant. There were no interactions involving tDCS polarity and gender for negative affect scores (all Fs <0.2, all p’s>.7, all η_p_^2^<0.01). Bayesian analysis showed that the preferred model was the main effect of gender for positive affect (BF = 0.6) and the main effects of both time and gender for negative affect (BF = 1.2), but the evidence for these models was weak. A model of the interaction between time, gender, and tDCS polarity supported the null hypothesis for both positive affect (BF <0.01) and negative affect (BF = 0.01).

For the filler task, there were no effects of gender on RTs (all Fs <1.6, all p’s>.2, all η_p_^2^<0.1) or accuracy (all Fs <2.3, all p’s>.1, all η_p_^2^<0.13), and Bayesian analysis showed that the preferred model was still the interaction between stimulus location and response location (BF = 43).

## Discussion

The polarity of tDCS to DLPFC had no effect on the speed or accuracy of memory retrieval of emotional images and did not influence positive or negative affect, as measured by the PANAS questionnaire. The current density applied to DLPFC was relatively high in comparison to other tDCS studies, therefore we can assume that the electric field induced during tDCS did affect cortical neuronal activity in the DLPFC regions underlying the electrodes. Indeed, the polarity of tDCS to left and right DLPFC modulated performance on the stimulus-response compatibility task which was performed during the application of tDCS, suggesting that neural activity was successfully altered by tDCS, although note that this is based on an exploratory data analysis and further work using larger sample sizes will be required to corroborate this finding. Previous work has shown that the excitatory or inhibitory effects of tDCS to motor cortex last for up to 40 minutes [7]; therefore it is probable that the physiological changes produced by tDCS in the present study remained for the duration of the memory retrieval task and the PANAS questionnaire. Note, however, that the time course of tDCS aftereffects has not been examined for tDCS to prefrontal brain regions.

Negative affect decreased after tDCS, which could be interpreted as an overall polarity-independent effect of tDCS on mood. However, further examination of the data showed that this was largely due to decreased scores on the “nervous” item after tDCS. A recent meta-analysis showed that, in the majority of studies which have examined the effect of tDCS on cognitive tasks, the anode and cathode do not produce similar effects on performance [48]. The analysis found only two studies in which the effects of anodal and cathodal tDCS were equivalent. However, in one of these studies equal effects of cathodal and anodal stimulation were only observed for drug users [49] while the polarities had different effects (in the same task) for healthy individuals [50]. The other study to find similar effects of anodal and cathodal stimulation used a type of time-varied tDCS [51], which may not be comparable to the standard procedure. Therefore, we consider it unlikely that both tDCS polarities had a similar effect on emotional memory retrieval in the present study.

The finding that prefrontal tDCS does not influence self-reported emotional state in healthy individuals is consistent with the majority of previous research. Marshall et al. [52] observed an increase in mood, measured by self-report, after anodal tDCS was applied intermittently to both left *and* right DLPFC; however this result was not replicated in subsequent work [18], [19]. Several other studies have found no effects of prefrontal tDCS on subjective measures of emotional state [20]–[24].

However, studies using indirect behavioural measures of emotional processing have shown inconsistent results. Recent work has shown that the polarity of tDCS to left DLPFC did not significantly influence reaction time to indicate the location of emotional faces [22]. Two studies have found that participants rated aversive images as less unpleasant during and after anodal tDCS to left DLPFC compared to baseline [23], [25]. Conversely, other work has found that participants rated negative emotional images as more unpleasant after anodal tDCS to left DLPFC compared to sham tDCS, and this was associated with a decrease in EEG alpha power over left DLPFC [21]. Cathodal tDCS to left DLPFC had no effects on ratings of emotional images [21], [23].

There have also been inconsistent findings from studies that have examined the effects of prefrontal tDCS on autonomic arousal in healthy volunteers. Anodal tDCS to DLPFC has been reported to have no effect on skin conductance responses when viewing emotional images [20]. However, recent work has shown that anodal tDCS to left DLPFC produced a decrease in cortisol levels and higher heart rate variability when viewing negative emotional images relative to cathodal or sham tDCS, and these effects may reflect suppression of the hypothalamic-pituitary-adrenal and sympatho-adreno-medullary systems, which are dysfunctional in mood disorders [53]. In both studies subjective emotional state was not modulated by tDCS.

Of most relevance to the present study, Penolazzi et al. found that anodal tDCS of left DLPFC with the cathode over right DLPFC improved free memory recall of unpleasant images, relative to the opposite polarity (i.e. cathode over left DLPFC) which was found to improve memory recall of pleasant images [26]. Note that this is the opposite effect to what would be expected based on neuropsychological findings. That is, stroke patients with damage to left prefrontal regions are more likely to experience depression, whereas patients with right prefrontal damage may display symptoms of euphoria, such as pathological laughter [54]–[56], which suggests that reducing left prefrontal activity (with cathodal tDCS) should reduce mood and increase memory recall of unpleasant items while enhancing left prefrontal activity should enhance mood and increase memory recall of pleasant items. However, Penolazzi et al. applied tDCS prior to encoding the images, making it difficult to determine whether the tDCS affected visual processing, memory encoding, or memory retrieval of the images. By contrast, the present study specifically examined the effect of tDCS on memory retrieval by applying tDCS after the encoding phase and before the retrieval phase. It is therefore possible that tDCS to DLPFC affects encoding of emotional stimuli, but may not affect recognition. Furthermore, the use of larger electrodes in Penolazzi et al.’s study, that were placed slightly more posterior compared to most other studies using prefrontal tDCS, may have directed some of the current to regions of temporal cortex, which is thought to be involved in memory encoding [57].

It is possible that anodal tDCS of left DLPFC only improves mood in depressed patients. That is, tDCS may be more effective on pathological patterns of neural activity. In support of this idea, repetitive transcranial magnetic stimulation (TMS) of prefrontal cortex has also been reported to improve mood in depressed patients [58]–[63], and a recent meta-analysis suggests that this benefit is clinically meaningful [64], whereas results concerning mood changes following TMS in healthy individuals have been inconclusive, with few studies showing improvement in mood [65] (but see [66]) and others finding no significant effects [67]–[70]. Furthermore, consecutive sessions of tDCS may be necessary to produce effects on mood; studies using anodal tDCS to left DLPFC to treat depression have applied tDCS daily for several consecutive days, whereas the present study only gave one session for each polarity. Other work suggests that the effects of brain stimulation techniques such as TMS and tDCS depend on the initial neural activation state, and may be more effective on the least active neurons [71], supporting the idea that tDCS may be more effective in depressed patients than in healthy individuals.

However, as noted previously, sham-controlled clinical studies of tDCS to DLPFC as a treatment for depression have shown inconsistent results [9]–[15]. Furthermore, meta-analyses of these sham-controlled studies have also found inconsistent results, with one meta-analysis reporting that active tDCS was more effective than sham tDCS for the reduction of depression severity [72] and another meta-analysis reporting no significant difference between active and sham tDCS in response and remission rates [73]. There was also a significant amount of heterogeneity in outcome measures between the studies, which may reflect variability in patient characteristics and stimulation parameters [72]. For example, participants in three of the studies [9]–[11] were not on antidepressant medication, whereas the other studies contained a mixture of antidepressant-free patients and patients on stable antidepressant treatment. The studies also differed in stimulation parameters such as the number of sessions (between 5 and 15) and the spacing of the sessions. Notably, only four of the studies showed successful blinding (i.e. patients were unable to guess whether they were in the active or sham condition) [12]–[15], [73], and of these studies only one [12] found that tDCS improved symptoms of depression. The other studies which showed effects of tDCS on depression had very small numbers of patients [5]–[10] in each group and did not report the integrity of blinding procedures [9]–[11]. It remains unclear how many consecutive sessions of tDCS are necessary to produce effects on mood, or whether any effects can be observed after a single session of tDCS, and further work using larger sample sizes is needed to determine the efficacy of this technique in depressed patients.

Participants’ performance on the stimulus-response compatibility task during the application of tDCS was modulated by the polarity of tDCS to left and right DLPFC, and this effect was dependent on participants’ motivational state in relation to the experiment. That is, highly motivated participants were faster to respond to targets on the right when the anode was over left DLPFC, whereas they were faster to respond to targets on the left when the anode was over right DLPFC. This may reflect the role of DLPFC in cognitive control [74] and spatial attention [75]. By contrast, the performance of participants with low levels of motivation was not influenced by the polarity of tDCS. This is consistent with previous work showing that the effects of prefrontal tDCS depend on participants’ motivational state [27], [28]. Indeed, prefrontal cortex has been shown to play an important role in motivating behaviour and integrating motivation and cognitive control processes [76], [77]. Therefore, in the context of research showing that the effects of brain stimulation may depend on the initial activation state of neurons [71], it is logical to assume that both motivational state and the effects of tDCS may depend on prior levels of prefrontal activity.

In conclusion, we have shown that altering the balance of activity between left and right DLPFC does not change the mood state of healthy individuals. It is possible that consecutive sessions of tDCS are required to produce mood changes, or that prefrontal tDCS is only effective on pathological patterns of neural activity. The finding that the effects of tDCS on a cognitive task are dependent on participants’ motivational state supports other work showing that the efficacy of brain stimulation techniques is influenced by the prior activation state of neurons. Future work needs to determine whether consecutive sessions of tDCS can alter mood in healthy individuals and the extent to which effects of prefrontal tDCS depend on the initial activation state of neurons. In particular, we suggest that the effects of tDCS on cognition and behaviour could be maximised by placing participants in a receptive state prior to stimulation.
